# A shared neural network for emotional expression and perception: an anatomical study in the macaque monkey

**DOI:** 10.3389/fnbeh.2015.00243

**Published:** 2015-09-24

**Authors:** Ahmad Jezzini, Stefano Rozzi, Elena Borra, Vittorio Gallese, Fausto Caruana, Marzio Gerbella

**Affiliations:** ^1^Department of Anatomy and Neurobiology, Washington University in St. LouisSt. Louis, MO, USA; ^2^Department of Neuroscience, University of ParmaParma, Italy; ^3^Brain Center for Social and Motor Cognition, Istituto Italiano di TecnologiaParma, Italy

**Keywords:** insula, emotion expression, emotion perception, lip-smacking, affiliative field, interoception

## Abstract

Over the past two decades, the insula has been described as the sensory “interoceptive cortex”. As a consequence, human brain imaging studies have focused on its role in the sensory perception of emotions. However, evidence from neurophysiological studies in non-human primates have shown that the insula is also involved in generating emotional and communicative facial expressions. In particular, a recent study demonstrated that electrical stimulation of the mid-ventral sector of the insula evoked affiliative facial expressions. The present study aimed to describe the cortical connections of this “affiliative field”. To this aim, we identified the region with electrical stimulation and injected neural tracers to label incoming and outgoing projections. Our results show that the insular field underlying emotional expression is part of a network involving specific frontal, cingulate, temporal, and parietal areas, as well as the amygdala, the basal ganglia, and thalamus, indicating that this sector of the insula is a site of integration of motor, emotional, sensory and social information. Together with our previous functional studies, this result challenges the classic view of the insula as a multisensory area merely reflecting bodily and internal visceral states. In contrast, it supports an alternative perspective; that the emotional responses classically attributed to the insular cortex are endowed with an enactive component intrinsic to each social and emotional behavior.

## Introduction

According to the classical view of affective neuroscience, emotion and its expression are two separate phenomena. This perspective considers emotion as a sensation either preceding or consequent to its expression, and that the behavioral outcome of an emotion would be independent from the emotion itself. Systems neuroscience has long made the assumption that emotional recognition and expression are governed by distinct neural circuits. Accordingly, the expression of emotion is thought to be dependent upon frontal motor areas, while the feeling of emotion relies on a series of perceptual cortical and subcortical regions. In line with this dichotomy, the insular cortex has been described as a sensory cortex involved in processing the perceptual aspects of emotion (Craig, 2002; Damasio, 2003a,b; Critchley et al., 2004). More specifically, it has been suggested that the insula is an interoceptive cortex that integrates homeostatic, visceral, nociceptive, and somatosensory inputs (Craig, 2002). Single neuron studies have supported this view, showing that posterior sectors of the monkey insula encode somatosensory, nociceptive and auditory information (Robinson and Burton, 1980; Schneider et al., 1993; Zhang et al., 1998; Remedios et al., 2009), while neurons recorded from the more anterior insular sectors encode gustatory stimuli (Yaxley et al., 1990; Verhagen et al., 2004). Little is known about the ventralmost portion of the insula.

The sensory interpretation of the functional role of the insula, albeit supported by human imaging data (Kelly et al., 2012), is at odds with results of classic stimulation studies in non-human primates. These studies aimed to assess the role of the insular cortex in generating behavioral responses and showed that electrical stimulation of the insula in deeply anesthetized monkeys evoked a series of autonomic reactions, including respiratory and vascular responses (Kaada et al., 1949; Hoffman and Rasmussen, 1953; Showers and Lauer, 1961) as well as orofacial motor responses (Frontera, 1956; Showers and Lauer, 1961).

Recent studies from our group further investigated this issue by performing electrical intracortical microstimulation (ICMS) in awake macaque monkeys (Caruana et al., 2011; Jezzini et al., 2012). We found that the application of prolonged ICMS to different insular sectors elicited a variety of overt motor behaviors ranging from ingestive and mouth-related responses to sensorimotor responses involving the upper and lower limbs. Stimulations along the dorso-ventral axis showed a shift from non-emotional sensorimotor behaviors, elicited in proximity to the frontoparietal operculum, to emotional and social interaction behaviors, elicited in proximity to the temporal operculum. In particular, electrical stimulation of the anterior sector of the insula evoked disgust-related and ingestive behaviors, while stimulation of its mid-dorsal sector most often produced forelimb movements, and stimulation to the mid-ventral sector evoked “lip-smacking” behavior. The latter behavior is an affiliative and communicative monkey facial expression with a reassuring function, normally preluding the tendency to approach a conspecific. The presence of a human homologue of the affiliative field in the ventral insula has been suggested by recent data demonstrating that patients who undergo insular resection show poor ability to recognize facial expressions and in particular, those related to the expressions of positive emotions such as happy and surprised faces (Boucher et al., 2015). In striking accord with our data, the highest overlap of resections has been found at the level of the ventral sector of the insula. In addition to the motor component of the response, the evoked lip-smacking behavior was accompanied by a decrease of the animal’s heart rate, suggesting a control of the parasympathetic nervous system. Remarkably, this behavior was only evocable during ICMS when eye contact between the monkey and experimenter was established. This suggested that the social context imposed by the mutual gaze is crucial in modulating the response threshold of the insula.

The behavioral responses evoked by prolonged stimulation are typically interpreted as the effect of a transynaptic spread of signal through a network of anatomically connected areas (Graziano et al., 2002). Accordingly, we hypothesized that the behavioral outcome of our stimulations was most likely due to the recruitment of a network of areas encoding sensory, social, motor and visceromotor information. These areas, according to this hypothesis, would be anatomically connected to the identified affiliative field of the mid-ventral insula. The aim of the present study was to demonstrate the existence of this functional network, and to define its neuroanatomical extent. We injected retrograde and anterograde neural tracers in the affiliative field of the insula, as functionally identified by ICMS, and plotted labeled cells and axonal projection fields. To determine whether the observed anatomical circuit is unique to the affiliative field: (1) we compared the connectional pattern obtained after injections into the affiliative field with that of injections in the adjacent disgust/ingestive insular field and (2) we identified an insular region involved in sensorimotor forelimb control by analyzing the insular labeling after injections into two hand-related motor fields of the parietal and premotor cortex (area PFG and F5, respectively). With these results, we propose an anatomical network at the basis of emotional expression and discuss the possible functional role of its nodes in lip-smacking behavior.

## Materials and Methods

Two male rhesus monkeys (Macaca mulatta), MK1 and MK2, weighing 7 and 11 kg, respectively, were employed in this study. These monkeys were previously used in electrophysiological experiments (Caruana et al., 2011; Jezzini et al., 2012) to functionally map various insular regions. Additional data from one monkey (M2), already partially presented in a previous neurophysiological work (Bonini et al., 2010), were here reanalyzed for the purpose of the present study. The animal handling, as well as surgical and experimental procedures, complied with the European guidelines (86/609/EEC 2003/65/EC Directives and 2010/63/EU) and Italian laws in force of the care and use of laboratory animals. Further, experiments were approved by the Veterinarian Animal Care and Use Committee of the University of Parma (Prot. 78/12 17/07/2012) and authorized by the Italian Health Ministry (D.M. 294/2012-C, 11/12/2012).

### Tracer Injections and Histological Procedures

**Figure 1:**
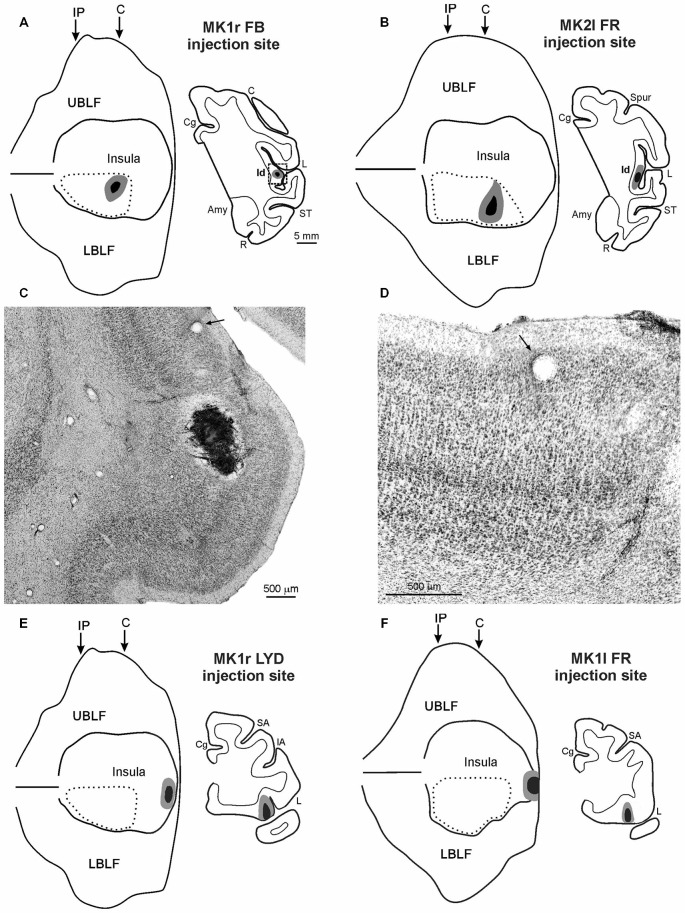
**(A,B,E,F)** Injection sites shown in 2D reconstructions of the LF and in drawings of the sections. Each 2D reconstruction was aligned to correspond with the middle of the insula; the continuous lines mark the lips of the sulcus, the border of the insula with the upper and lower bank of the sulcus, and the fundus. The dashed lines indicate the region containing all the microstimulations that evoked an affiliative response. Arrows mark the levels of the rostral tip of the intraparietal sulcus (IP) and of the rostralmost level of the central sulcus (C). The location of each tracer injection is shown as a black zone corresponding to the core, surrounded by a gray zone corresponding to the halo. **(C)** Low-power photomicrograph of Nissl-stained coronal section shown in **(A)**. The dashed box in the section drawing indicates the location of the photomicrograph. **(D)** Higher magnification view of the photomicrograph shown in **(C)**. Arrows in **(C,D)** indicate the same blood vessel. Scale bars in **(A)** apply also to **(B,E,F)**. Amy, amygdala; C, central sulcus; Cg, cingulate sulcus; IA, inferior arcuate sulcus; L, lateral fissure; LBLF, lower bank of the LF; R, rhinal sulcus; SA, superior arcuate sulcus; ST, superior temporal sulcus; UBLF, upper bank of the LF.

The choice of the injection sites in the insular cortex was based on data obtained by electrical stimulations performed in the functional mapping study of the insula and the perisylvian regions (Caruana et al., 2011; Jezzini et al., 2012). At the end of the physiological experiments, neural tracers were injected at specific coordinates of the recording grid through the intact dura. In both monkeys, we injected at the core of the regions from which affiliative or disgust-related behaviors were evoked (Figure 1), as confirmed by a preoperative stimulation session (using the same parameters described in Caruana et al., 2011; Jezzini et al., 2012). The depths of the injection sites were chosen on the basis of these data and the presence of neuronal activity confirmed by a recording session performed immediately before injecting the tracers. During the injection, the location of the syringe tip was continuously monitored by means of ultra-sound imaging (see Caruana et al., 2011; Jezzini et al., 2012).

In M2 neural tracers were injected in the PFG and F5 sectors where hand-grasping motor neurons had been recorded (see Bonini et al., 2010). A recording session performed immediately before the tracer injection confirmed the presence of reliable neural activity and properties coherent with those previously found during the electrophysiological experiment.

Each monkey was anesthetized (Ketamine, 5 mg/kg i.m. and Medetomidine, 0.08–0.1 mg/kg i.m.) and tracers were slowly pressure injected at the desired depth through a Hamilton microsyringe (Reno, NV, USA). In the right hemisphere of MK1, we used the retrograde tracer Fast Blue (FB, 3% in distilled water, Drilling Plastics GmbH, Breuberg, Germany) in the affiliative field, and the retro-anterograde tracer Lucifer Yellow (10,000 MW, LYD, 10% phosphate buffer 0.1 M, pH 7.4; Invitrogen-Molecular Probes, Eugene, OR, USA) in the disgust/ingestive field. In the left hemisphere of MK1, we used the retro-anterograde tracer Dextran conjugated with tetramethylrhodamine (10,000 MW, Fluoro-Ruby, FR, 10% phosphate buffer 0.1 M, pH 7.4; Invitrogen-Molecular Probes, Eugene, OR, USA) in the disgust/ingestive field. In the left hemisphere of MK2, we injected FR and LYD in the affiliative field. In the right hemisphere of M2 we injected the B subunit of the cholera toxin conjugated with Alexa 594 (CTBr) or Alexa 488 (CTBg 1% in phosphate-buffered saline; Molecular Probes, Eugen, OR, USA) in PFG and F5, respectively. The details of the injections are provided in Table 1.

**Table 1:** **Cases, hemispheres, localization of the injection sites, and tracers employed in the experiments**.

Cases	Left/Right	Injected Field	Tracer	Amount
MK1	R	Insula: affiliative	FB 3%	1 × 0.3 μl
	R	Insula: disgust/ingestive	LYD 10%	1 × 1 μl
	L	Insula: disgust/ingestive	FR 10%	1 × 1 μl
MK2	L	Insula: affiliative	FR 10%	1 × 1 μl
	L	Insula: affiliative	LYD 10%	1 × 1 μl
M2	R	F5: hand	CTBg 1%	1 × 1 μl
	R	PFG: hand	CTBr 1%	1 × 1 μl

Ten days before sacrificing each animal, electrolitic lesions (10 μA cathodic pulse for 10 s) were performed at known coordinates of the recorded region in order to match functional and anatomical data. After appropriate survival periods (28 days for FR and LYD and 14 days for FB, CTBr and CTBg) each animal was deeply anesthetized with an overdose of sodium thiopental and transcardially perfused with consecutive solutions of saline, 3.5% paraformaldehyde, and 5% glycerol, prepared in 0.1 M phosphate buffer at pH 7.4. Each brain was then blocked coronally on a stereotaxic apparatus, removed from the skull, photographed, and placed in 10% buffered glycerol for 3 days, followed by 20% buffered glycerol for 4 days. Finally, they were cut frozen into coronal sections of 60 μm thickness. For the visualization of fluorescent tracers FB, CTBr and CTBg, every fifth section was mounted and coverslipped for fluorescent microscopy. In Case MK1 and MK2, one series of each fifth cut section was processed to visualize antero-retrograde tracers FR or LYD, using the following protocol. After inactivation of the endogenous peroxidase (methanol: hydrogen peroxide = 4:1), selected sections were incubated for 72 h at 4°C in a primary antibody solution of rabbit anti-FR or anti-LYD (1:3000; Invitrogen) in 0.3% Triton, 5% normal goat serum in PBS, and then incubated in biotinylated secondary antibody (1:200, Vector Laboratories, Burlingame, CA, USA) in 0.3% Triton, 5% normal goat serum in PBS. Finally, FR or LYD labeling was visualized using the Vectastain ABC kit (Vector) and the Vector SG peroxidase substrate kit (SK- 4700, Vector) as a chromogen. In all cases, one series of each fifth section was stained by Nissl method (0.1% thionin in 0.1 M acetate buffer, pH 3.7) for cytoarchitectonic analysis and for the identification of the electrolytic lesions.

### Data Analysis

The criteria used for the definition of the CTBr, CTBg, FB, FR, and LYD injection sites and labeling have been described in earlier studies (Luppino et al., 2003; Rozzi et al., 2006; Gerbella et al., 2011). The distribution of retrograde and anterograde cortical labeling was analyzed in sections every 300 μm and plotted in sections every 600 μm, together with the outer and inner cortical borders, using a custom computer-based charting system. The distribution of labeling in the lateral fissure (LF) and in the superior temporal sulcus (STS) was visualized in 2D reconstructions obtained using the same software, as follows (for more details, see Matelli et al., 1998). In each plotted section, the cortical region of interest was unfolded at the level of a virtual line running approximately along the border between layers III and IV. The unfolded sections were then aligned, and the labeling was distributed along the space between the two consecutively plotted sections (600 μm). Sections through the LF were aligned to correspond with the middle of the insula and those through the STS to correspond with the fundus and the middle of the floor. The criteria and maps used for the areal attribution of the labeling were similar to those used in previous studies (Rozzi et al., 2006; Gerbella et al., 2007, 2010, 2011). Specifically, the prefrontal cortex, including the orbitofrontal cortex, was subdivided according to Carmichael and Price (1994), except for the ventrolateral prefrontal (VLPF) cortex, which was subdivided according to Gerbella et al. (2007). The labeling was attributed to the agranular frontal and cingulate areas according to architectonic criteria previously described (Matelli et al., 1985, 1991; Belmalih et al., 2009). The attribution of the labeling to the frontal opercular areas was made according to the architectonic studies of Roberts and Akert (1963), Jones and Burton (1976), and Cipolloni and Pandya (1999), and according to recent architectonic and connectional data from Belmalih et al. (2009) and Gerbella et al. (2014). For the parietal operculum, we matched our data with the functional maps of the SII region by Fitzgerald et al. (2004). The lower bank of the STS was subdivided according to Seltzer and Pandya (1978). Finally, the insular cortex was subdivided according to Mesulam and Mufson (1982). The distribution of anterograde labeling in the ipsilateral basal ganglia was analyzed in sections every 300 μm in all of the cases. The projection fields in the basal ganglia were typically organized in patches of very dense labeled terminals, surrounded by less densely labeled zones. To obtain faithful reproductions of this labeling distribution, as in other studies (Parthasarathy et al., 1992; Calzavara et al., 2007; Borra et al., 2015), the distribution of the observed projection fields was visualized by extracting the labeling from digitalized photographs taken with a 10× objective. We used Adobe Photoshop (Adobe Systems Incorporated, San Jose, CA, USA) on each image to first outline the basal ganglia and adjacent structures in separate digital layers. Then, striatal projection fields were selected and converted into black-and-white images by applying a threshold appropriate to extract the labeling, stained in black or blue, from the lighter background. Comparison with the original image ensured that the labeling was accurately extracted.

Amygdalar and thalamic labeled cells were plotted in sections every 300 μm together with the outline of the ventricles and of blood vessels, using the aforementioned computer-based charting system. Borders of thalamic and amygdalar nuclei, defined in adjacent Nissl-stained sections, were then superimposed on the plots of labeled cells, using the outline of the ventricles and of blood vessels, with the aid of a microprojector and a camera lucida. The borders of the thalamic nuclei were primarily defined according to the cytoarchitectonic criteria and the nomenclature used by Olszewski (1952) and the amygdalar complex was subdivided according to the criteria described by Amaral et al. (2003).

## Results

### Insular Injection Sites Location

#### Injections in the Affiliative Field of the Insula

The injection sites of FB, in the right hemisphere of MK1, and of FR, in the left hemisphere of MK2, are shown in Figures 1A,B. The injection site of LYD in the left hemisphere of MK2, not shown, is located very close to the position of the FR injection. The injection sites were located in the mid-ventral part of the insula, in the sector in which ICMS evoked affiliative responses such as lip-smacking. The cytoarchitectonic features of the cortex surrounding the injection sites indicate that they are located within the dysgranular insula, as defined by Mesulam and Mufson (1982); see Figures 1C,D and more recently by Gallay et al. (2012) and Evrard et al. (2014). FB injection sites of MK1 and FR injection site of MK2 are completely restricted to the gray cortical matter and involve the entire cortical thickness whereas the LYD injection site of MK2 partially spreads into the claustrum, thus the following connectional data and figures will mainly focus on the former two injections. Note, however, that the MK2 LYD injection produced a very similar pattern of retrograde and the anterograde labeling to that of the other injections.

#### Injections in the Disgust/Ingestive Field of the Insula

The injection sites of LYD, in the right hemisphere of MK1, and of FR, in the left hemisphere of MK1, are shown in Figures 1E,F. They are located in the sector in which ICMS evoked disgust-related behaviors such as spitting food, throwing it away or retching, accompanied with a bradycardic effect, or ingestive behaviors such as chewing, mouthing and swallowing. The LYD injection site is confined to the anteriormost sector of the insula, while the FR injection is larger, and partially spreads into the adjacent orbital cortex. Despite the spread of the FR injection site, its resulting pattern of labeling is quite similar to that of the LYD injection. The architectonic features of the cortex surrounding the injection sites indicate that they are located within the agranular insula, according to the criteria defined by Mesulam and Mufson (1982), Carmichael and Price (1994), Gallay et al. (2012), and Evrard et al. (2014).

### Connections of the Affiliative Field of the Insula

#### Cortical Connections

**Figure 2:**
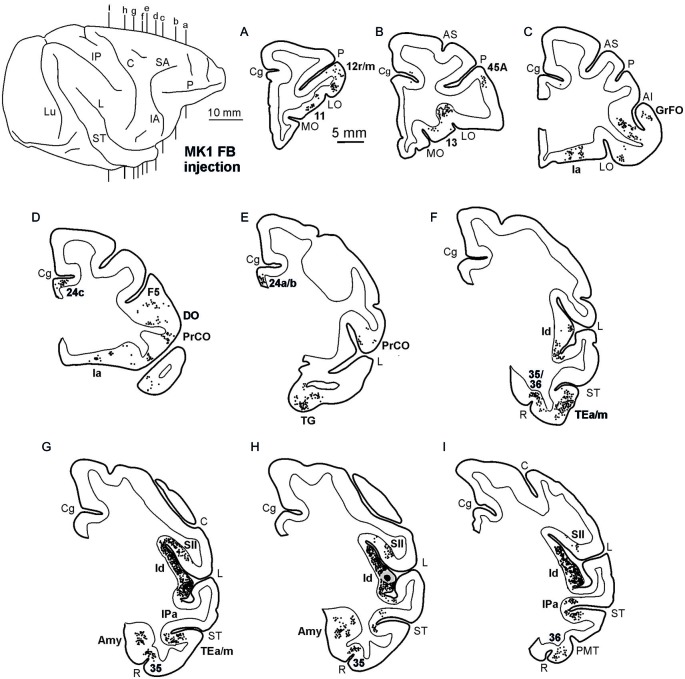
**Distribution of the retrograde labeling observed in Case MK1, shown in drawings of representative coronal sections.** Sections are shown in a rostral to caudal order **(A–I)**. Each circle corresponds to one labeled neuron and the injection sites are shown as a black zone corresponding to the core, surrounded by a gray zone corresponding to the halo. The dorsolateral view of the injected hemisphere in the upper left part of the figure shows the levels at which the sections were taken. AMT, anterior middle temporal sulcus; IO, inferior occipital sulcus; IP, intraparietal sulcus; LO, lateral orbital sulcus; Lu, lunate sulcus; MO, medial orbital sulcus; P, principal sulcus. Other abbreviations as in Figure 1.

**Figure 3:**
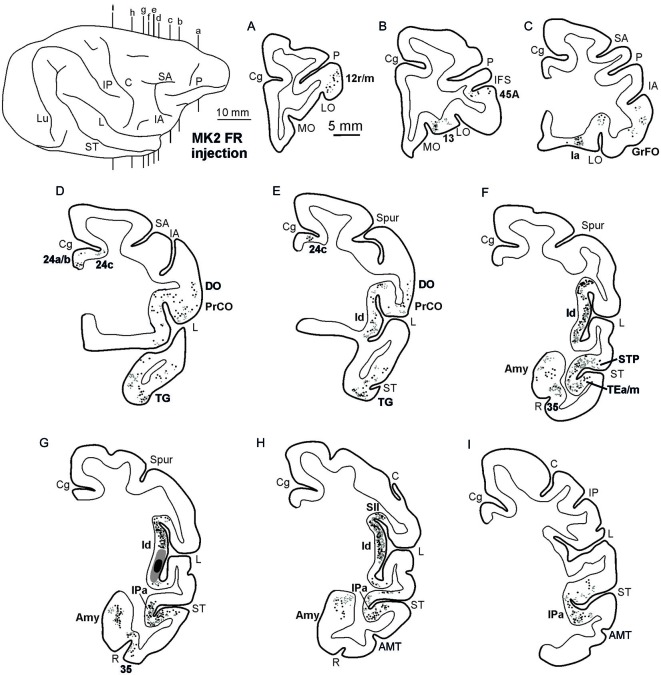
**Distribution of the retro-anterograde labeling observed in Case MK2, shown in drawings of representative coronal sections.** Sections are shown in a rostral to caudal order **(A–I)**. For the retrograde labeling, each black circle corresponds to one labeled neuron, and for the anterograde labeling, the gray circle density is proportional to the density of the observed labeled terminals (one gray circle is equivalent to about 15–25 labeled terminals). The dorsolateral view of the injected hemisphere in the upper left part of the figure shows the levels at which the sections were taken. Other conventions and abbreviations as in Figures 1,2.

**Figure 4:**
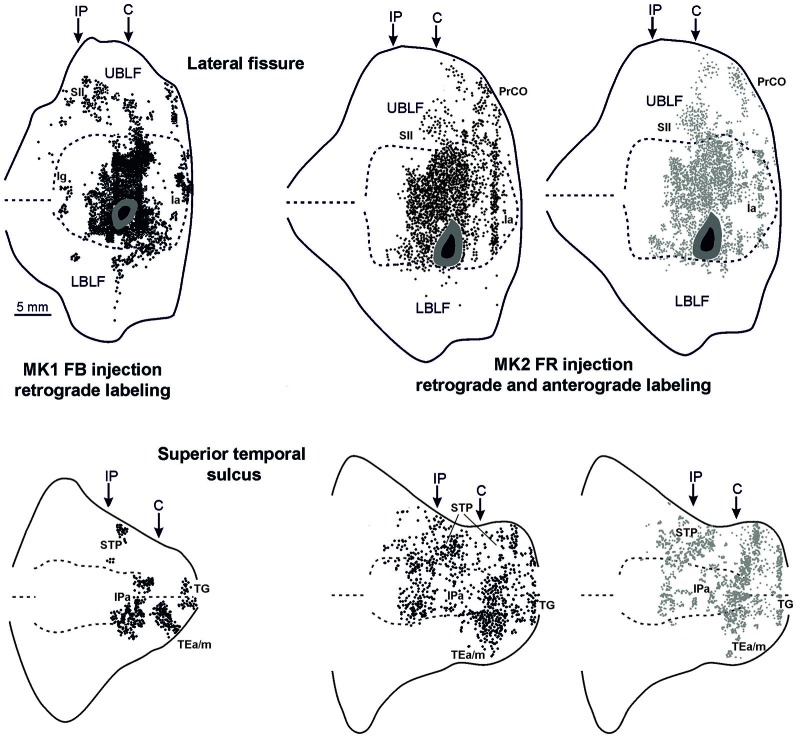
**Distribution of the retrograde and of the retro-anterograde labeling observed after injections of FB in case MK1 and of FR in MK2, respectively, shown in a 2D reconstruction of the LF (upper part) and of the STS (lower part).** For the retrograde labeling, each black circle corresponds to one labeled neuron, and for the anterograde labeling, the gray circle density is proportional tso the density of the observed labeled terminals (one gray circle is equivalent to about 15–25 labeled terminals). Each 2D reconstruction of the LF was aligned to correspond with the middle of the insula. The dashed lines indicate the fundus and the upper and lower edges of the floor, the continuous lines the lips of the sulcus. Each 2D reconstruction of the STS was aligned to correspond with the fundus and middle of the floor. The dashed lines indicate the fundus and the border of the insula with the upper and lower bank of the sulcus, the continuous lines the lips of the sulcus. Arrows mark the levels of the rostral tip of the intraparietal sulcus (IP) and of the rostral most level of the central sulcus (C). Other conventions and abbreviations as in Figures 1, 2.

Figures 2, Figures 3, 4 show the cortical distribution of labeled cells following the retrograde FB injection, as well as labeled cells and projection fields produced by the retro-anterograde FR injection. Within the insula, very dense labeling was found in the areas adjacent to the injection site, including the mid-dorsal (2G,H, Figures 3F–H, 4) and rostralmost regions of the insula (Ia; Figures 2C,D, 3C, 4), whereas the labeling in the posterior, granular insula was weak (Ig; Figure 4). In the frontal lobe, labeling was found in the orbitofrontal cortex, areas 12 m, 13, in the caudal half of area 11, and in the VLPF areas 12r and 46v/45A (Figures 2A,B, 3A,B). Outside the prefrontal cortex, retrogradely labeled neurons were found in the frontal opercular areas GrFO, PrCO, and DO (Figures 2D,E, 3D,E). In the medial wall, labeling was found in the mouth-related sector of the cingulate area 24c, extending to the caudal part of area 24a/b (Figures 2D,E, 3D,E). In the temporal cortex, labeled cells and terminals were observed in the rostral temporal pole (TG), in areas IPa and TEa/m of the STS, and in the entorhinal cortex, areas 35 and 36 (Figures 2E–I, 3E–I, 4, lower part). Clusters of labeled cells and terminals were found along the antero-posterior extent of the superior temporal polysensory (STP) area in MK2 and a mid-central part of STP in MK1 (Figure 4). Finally, in the parietal operculum, labeling was found, especially after FB injection, in the secondary somatosensory area SII (Figures 2G–I, 3G–I, 3G–I, 4, upper part).

#### Thalamic Connections

**Figure 5:**
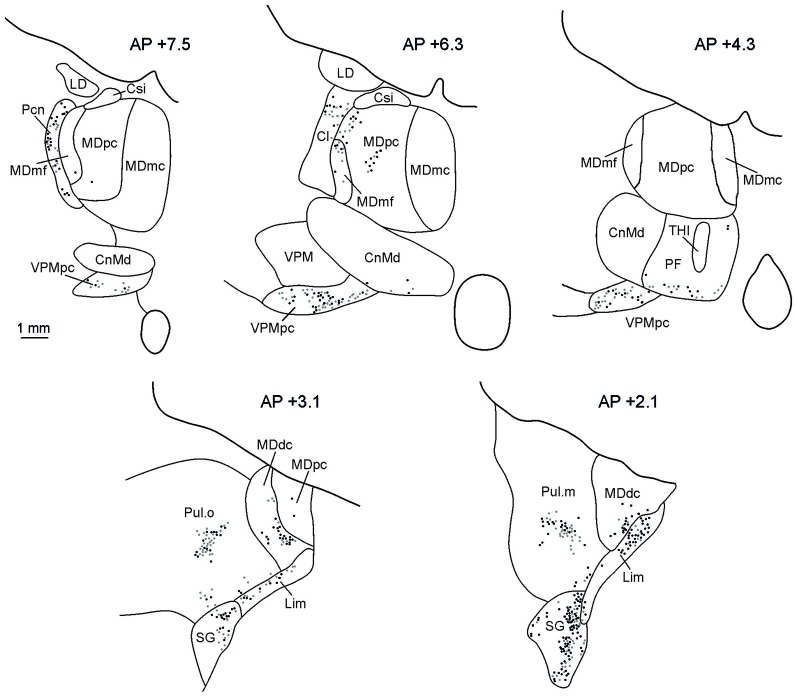
**Distribution of labeled thalamic neurons observed after FR injection in Case MK2.** The labeling is shown in drawings of coronal sections in rostral to caudal order selected at different AP levels according to the atlas of Olszewski (1952). Each dot corresponds to a single labeled neuron. Cl, central lateral nucleus; CnMd, centromedian nucleus; Csl, central superior lateral nucleus; LD, lateral dorsal nucleus; MDdc, mediodorsal nucleus, densocellular part; MDmc, mediodorsal nucleus, magnocellular part; MDmf, mediodorsal nucleus, multiform part; MDpc, mediodorsal nucleus, parvicellular part; Pcn, paracentral nucleus; Pf, parafascicular nucleus; SG, suprageniculate nucleus; THI, habenulointerpeduncular tract; VPM, ventral posterior medial nucleus; VPMpc, ventral posterior medial nucleus, parvicellular part.

The distributions of retrograde and anterograde labeling in the thalamus in all the cases were very similar. Figure 5 shows the retrograde and anterograde labeling at different rostro-caudal thalamic levels after FR injection in MK2. In rostral portions of the thalamus, labeled terminals and cells were observed mainly in the intralaminar nuclei (Paracentral nucleus, Pcn, and the central lateral nucleus, Cl), in the parvocellular subdivision of the mediodorsal nucleus (MDpc), in the parvocellular part of the ventral posterior medial nucleus (VPMpc), and in the ventral anterior nucleus (VA). More caudally, the labeling was virtually all located in the densocellular subdivision of MD (MDdc), in the oral and medial parts of the Pulvinar (Pul.O and Pul.M, respectively), in the suprageniculate nucleus (SG), and in the limitans nucleus (Lim).

#### Projections to the Striatum

**Figure 6:**
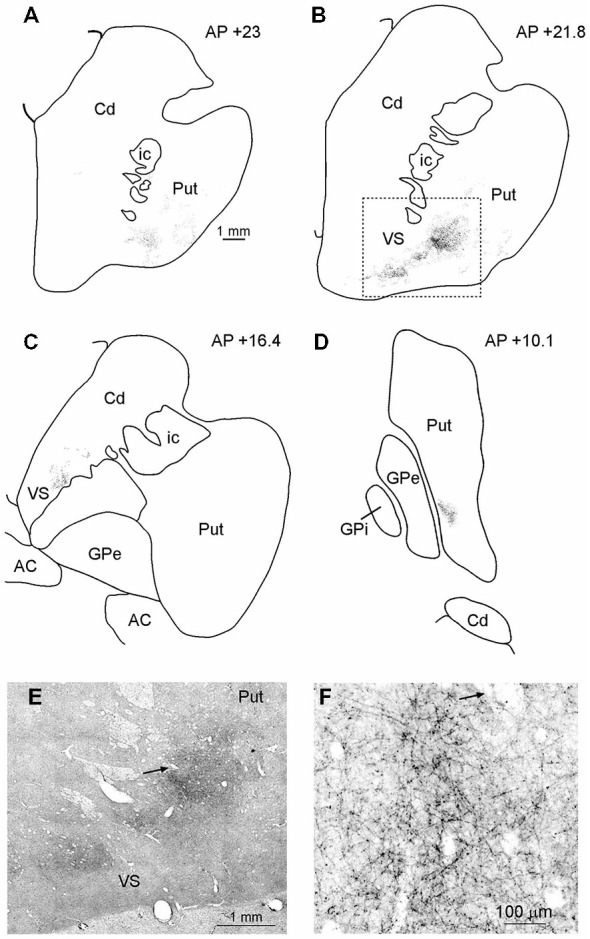
**(A–D)** Drawings of coronal sections through the striatum showing the distribution of the anterograde labeling observed after FR injection in Case MK2. The sections are shown in a rostral to caudal order **(A–D)**. **(E)** Low-power representative photomicrograph of the striatal anterograde labeling after FR injection in Case MK2; dashed boxes on the section **(B)** indicate the location of the photomicrograph. **(F)** Higher magnification view, taken from the photomicrograph shown in **(E)**. Arrows in **(E,F)** point to the same blood vessel. Scale bar in **(A)** applies also to **(B–D)**. Cd, caudate nucleus; GPe, external globus pallidus; GPi, internal globus pallidus; ic, internal capsule; Put, putamen; VS, ventral striatum.

In MK2, after both FR and LYD injections, labeled terminals were found in the striatum. In both cases, labeling was located at a similar location, although the pattern of labeling produced by the LYD injection was relatively sparser. Figure 6 shows the distribution of labeled terminals observed after FR injection. Rostral to the anterior commissure, the labeled terminals were mainly confined to the ventral part of the putamen and the ventral striatum (VS; Figures 6A–C,E,F). Caudal to the anterior commissure, the only putaminal sector labeled was found in a very ventral part of the posterior putamen (Figure 6D).

#### Connections with the Amygdala and Other Subcortical Structures

**Figure 7:**
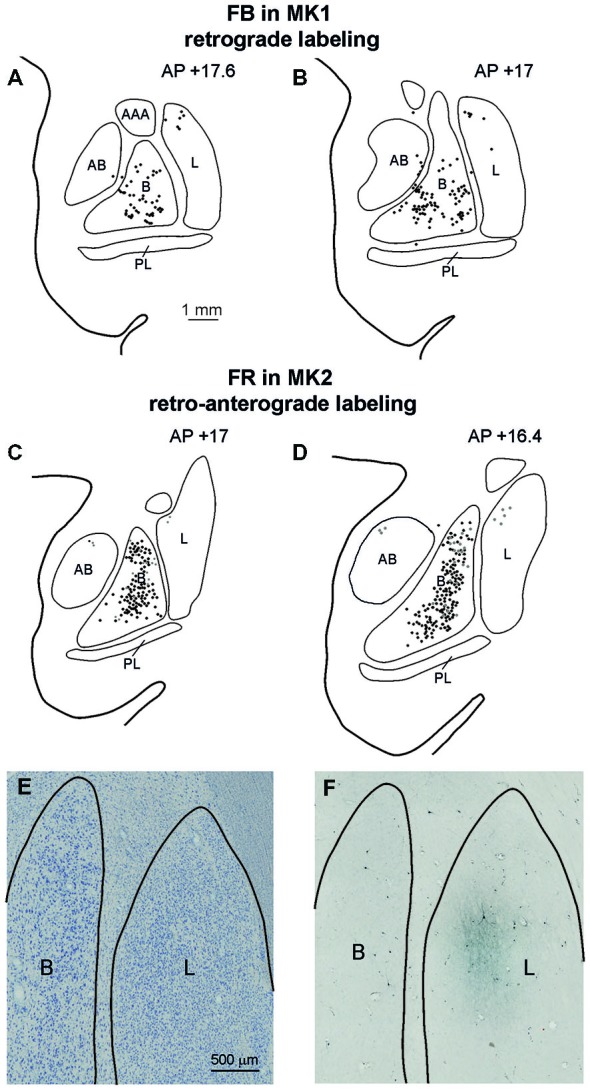
**(A–D)** Distribution of the amygdala retrograde and of the retro-anterograde labeling observed after injections of FB in case MK1 and of FR in MK2. For each case, the labeling is shown in two drawings of coronal sections, selected at different AP levels. Scale bar in **(A)** applies also to **(B–D)**. **(E,F)** Photomicrographs of a pair of adjacent coronal sections from Case MK2 FR, showing in **(E)** the distribution of retro- and anterograde labeling in the lateral and basal nucleus, compared with cytoarchitectonic subdivisions, shown in **(F)**. Scale bar in **(E)** applies also to **(F)**. AB, accessory basal nucleus; B, basal nucleus; L, lateral nucleus, PL, paralaminar nucleus. Conventions as in Figure 3.

Affiliative field injections in both cases produced a similar labeling pattern in the amygdala. Specifically, Figure 7 shows the amygdalar distribution of retrograde labeling following the FB injection (upper part, Figures 7A,B) and of the retro-anterograde labeling found after FR injection (middle part, Figures 7C,D; lower part, Figure 7F). Labeled cells were virtually all confined to the basolateral nucleus (B), while very few labeled cells were observed in the lateral nucleus (L) and in the accessory basal nucleus. The anterograde labeling observed after MK2 FR and LYD injections was localized within the B nucleus, where it overlapped with the retrograde labeling (Figures 7C,D), but also extended to the L nucleus (Figure 7F). Finally, some labeled neurons and terminals were observed in the posterior part of the lateral hypothalamic area and in the adjacent ventral tegmental area.

### Connections of the Disgust/Ingestive Field of the Insula

**Figure 8:**
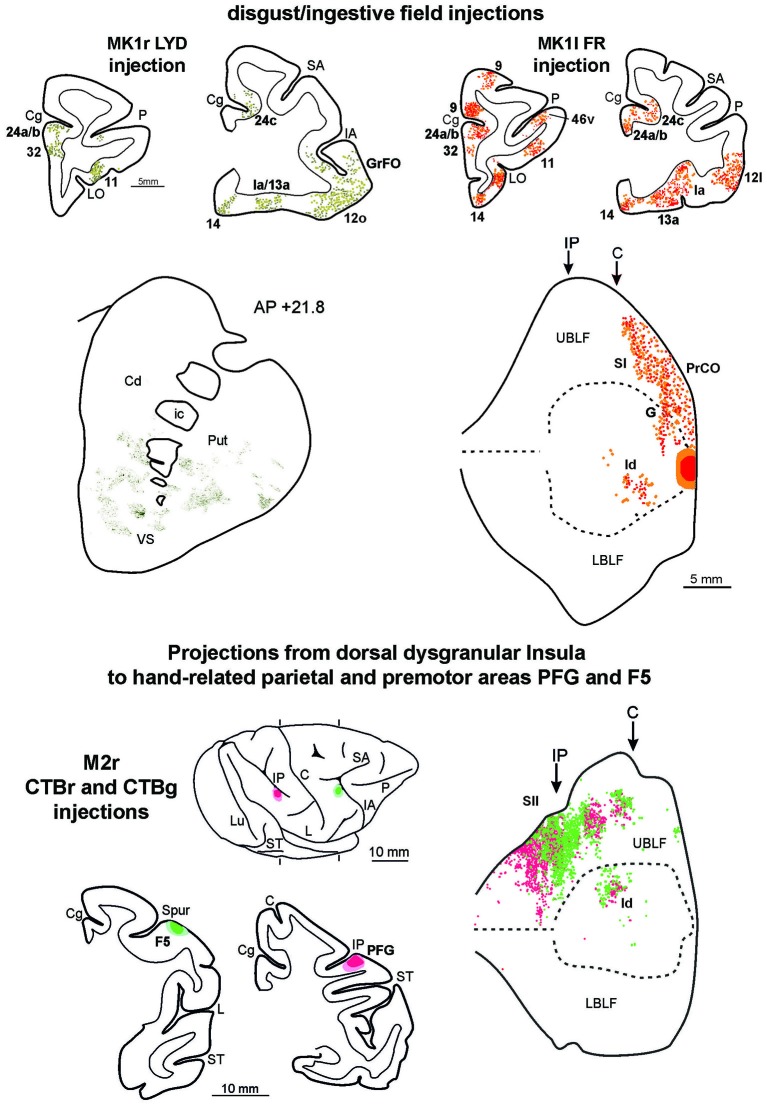
**Upper part left side: cortex and striatal representative coronal sections from MK1r LYD.** In the drawings of the cortex coronal sections, dark green squares and light green circles correspond to the anterograde and the retrograde labeling, respectively. In the digitalized microphotograph of the striatum the extracted anterograde labeling is shown in green. Upper part right side: representative coronal sections and unfolded view of the LF from MK1l FR. In the draws of the sections and in the unfolded view of the LF red and orange circles corresponds to the anterograde and the retrograde labeling, respectively. Lower part: the location of the injection sites in hand-related premotor and parietal areas, F5 and PFG, are shown in draws of the 2D reconstructions of the hemispheres and in coronal sections. In the unfolded view of the LF the F5 and the PFG retrograde labeling is shown by green and red circles, respectively. Conventions and abbreviations as in Figures 2, 3, 4, 6.

To compare the connectivity pattern of the affiliative field with that of the adjacent disgust/ingestive field, tracers were injected in the rostral part of the insula (see above). The results show that this cortical sector, within the insular cortex, is connected almost exclusively with the affiliative field (Figure 8, upper part). Furthermore, the disgust/ingestive field shares some connections with the affiliative one. Specifically, both injections in the disgust/ingestive field resulted in labeled cortical connections with orbitofrontal areas 11, 12 and 13, the frontal operculum (areas GrFO, PrCO and DO), the motor cingulate area 24c, the temporal pole (TG, slightly extending to the adjacent rostral inferotemporal cortex), and the entorhinal cortex. Other common connections are with subcortical structures, including the ventral part of the putamen, the VS, the basolateral and lateral nuclei of the amygdala, the posterior part of lateral hypothalamic area and the ventral tegmental area. For technical reasons concerning the histological processing of the thalamus in MK1 we cannot provide a complete description of the thalamocortical connections. However, the disgust/ingestive field has patterns of connection with other brain regions that set it apart from those of the affiliative field, shown in Figure 8 (upper part). In particular, in both cases, labeling was found close to the olfactory tract in areas 13a and 14, within the “olfactory neocortex” as defined by Carmichael and Price (1994), in the mesial prefrontal area 32, in the rostralmost part of cingulate area 24 (including rostral part of area 24a/b), in the anterior half of orbital area 11. In case MK1l FR, additional connections were found in area 9. Further characterizing connections after both the injections in the disgust/ingestive field were observed in the anterior part of the caudate nucleus and the accessory basal nucleus of the amygdala.

### Connections of the Dorsal Insula with Parietal and Premotor Hand-Related Fields, Indirect Data

Functional evidence from ICMS indicate that a specific sector of the insula, located just dorsal to the affiliative field, hosts a large hand-related field (see Figure 1A of Jezzini et al., 2012). In order to obtain anatomical evidence supporting the connectional distinctiveness of the affiliative field with respect to adjacent insular regions, we analyzed the distribution of the insular labeling found after injections of retrograde tracers in the sectors of parietal area PFG and premotor area F5 where hand-grasping motor neurons have been recorded (see Bonini et al., 2010). The results show that only the mid-dorsal part of the insula corresponding to the hand-related field projects to the hand-related parietal and premotor areas PFG and F5 (Figure 8, lower part).

## Discussion

### Connections of the Affiliative Field

**Figure 9:**
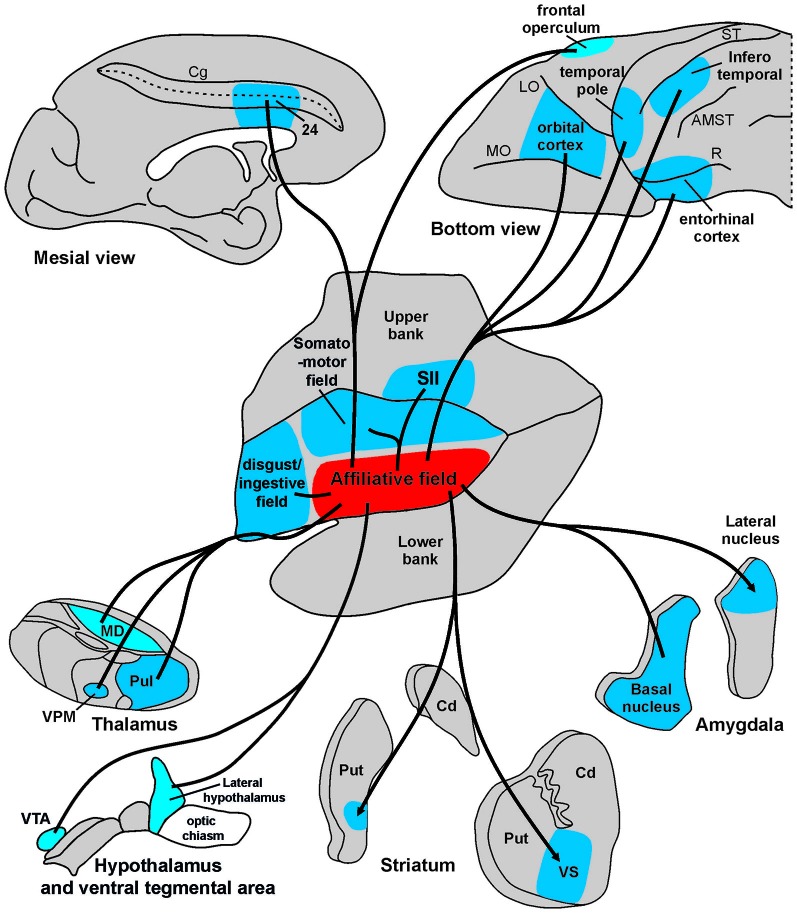
**Summary view of cortical and subcortical connections of the “affiliative field” of the insula.** Abbreviations as in Figures 1, 2, 5, 6.

The results of the present study show that the affiliative field of the ventral insula is anatomically connected with the adjacent rostral and dorsal insular regions and with a series of frontal, cingulate, parietal, and temporal areas, as well as with subcortical centers including the amygdala, the basal ganglia, the hypothalamus, and sensory-related thalamic nuclei (Figure 9). These results, in addition to confirming previous anatomical observations of insular connections (Mufson and Mesulam, 1982), largely extend our knowledge and allow us to link the underlying anatomical connections of a mid-ventral insular circuit to its functional role in the production of emotion-related facial expressions appropriate to a specific social context. Based on their functional properties, the cortical and subcortical districts involved in this network can be grouped in three main groups: visual, emotion-related, and sensory-motor regions. Each cortical or subcortical delineation of these groups is connected with the affiliative insular field and is possibly involved in controlling different aspects of social and emotional behavior.

#### Connections with Visual Regions

The present study showed a robust pattern of anatomical connections between the ventral insula and several subcortical (thalamic visual nuclei and ventral putamen) regions, as well as cortical high-order visual areas, including the temporal areas TE and IPa. These areas are known to be involved in processing visual information about eye direction, body orientation, facial expressions, and biological motion (Bruce et al., 1981; Rolls et al., 1982; Perrett et al., 1989; Puce et al., 1998; Pelphrey et al., 2003a,b; Moeller et al., 2008; Tsao and Livingstone, 2008), which are among the most relevant visual signals needed by an individual to interpret others’ behavior, and thus for processing social cues. This pathway provides a route through which visual information about social context can pass from the temporal cortex to the ventral insula. In particular, mutual gaze is known to trigger a stronger physiological arousal compared to other visual information, and to enhance attention to subsequent stimuli (Nichols and Champness, 1971; Kampe et al., 2001; Senju and Johnson, 2009). This could have a strong impact on insular neurons’ activity, possibly setting the intention to start communicative interactions necessary to manifest one’s emotional state to the other individual. This is in line with electrophysiological evidence (Caruana et al., 2011; Jezzini et al., 2012) showing that, unlike other behaviors elicited by ICMS of the insula, the affiliative ones (including lip-smacking) require, in addition to electrical stimulation, direct eye contact between subject and experimenter.

#### Connections with Emotion- and Memory-Related Regions

The affiliative field of the insular cortex is connected to several cortical areas and subcortical regions that could directly or indirectly provide it with the emotional significance of incoming sensory inputs. In particular, the anterior insula, the prefrontal cortex, the orbitofrontal cortex, the amygdala, the VS, the lateral hypothalamus, the ventral tegmental area, and the mediodorsal nucleus of the thalamus are regions involved in encoding the emotional aspects of sensory stimuli and integrating reward and memory with behavior (Oyoshi et al., 1996; Chikama et al., 1997; Barbas, 2007; Gothard et al., 2007; Grabenhorst and Rolls, 2011; Padoa-Schioppa and Cai, 2011; Jezzini et al., 2013). In addition, the affiliative field of the insular cortex is connected with the anterior temporal pole and the entorhinal cortex, which play a role in the maintenance of mnemonic representations (Nakamura and Kubota, 1995). These connections could represent the neural pathway through which information about salient experienced/emotional events and specific visual stimuli converge on the affiliative insula. Accordingly, it is interesting to remember that in our ICMS experiments (Caruana et al., 2011; Jezzini et al., 2012), memory related information regarding the identity of the experimenter and the associated emotional meaning of the visually perceived face allowed the release of a specific behavioral output.

These aforementioned visual and emotion-related areas connected to the affiliative field are also highly interconnected with one another, forming a robust circuit with the capacity to simultaneously process visual and emotional stimuli. This network appears to be involved in processing emotionally relevant cues by binding perceptual representations of sensory stimuli with emotional aspects of memorized experiences (Frank and Sabatinelli, 2014; Frank et al., 2014).

#### Connection with Sensory-Motor Regions

The ICMS of the affiliative insula elicits the production of facial emotional expressions such as lip-smacking (Caruana et al., 2011; Jezzini et al., 2012). Therefore, an important aim of the present study was to assess whether this insular sector is anatomically connected to the sensory-motor system. We found connections with two cortical regions involved in motor control of the face, one located in the cingulate cortex, the other on the lateral convexity of the frontal operculum.

The first region connected with the affiliative insula includes the anterior part of cingulate cortex known to be involved in the motor control of facial expressions (Morecraft et al., 1996, 2001, 2007; Gothard, 2014). The sector of the cingulate cortex connected with the “affiliative insula” likely corresponds to the face representation in area 24c, referred to as M3 by Morecraft and Van Hoesen (1992), who described the connections of this cortical field with the facial nucleus and the amygdala (Morecraft et al., 2007). In this respect, it has been recently shown that single neurons of both cingulate cortex, likely corresponding to area 24c, and the amygdala are active during the production of the lip-smacking behavior (Livneh et al., 2012). Note that both of these structures are also active during the observation of emotional expression produced by other individuals (Engen and Singer, 2013). This anterior cingulate-facial nucleus pathway could represent the principal motor output of a larger emotional network, also including the insular “affiliative field” and the amygdala, and may be responsible for the generation of emotional facial expressions appropriate to the perceived emotionally relevant stimuli. Recent evidence that electrical stimulation of the human anterior cingulate cortex elicits laughter and in most cases, also mirth and merriment (Caruana et al., 2015) further supports our view that the entire emotional network is involved in both emotional experience and expression.

The second motor region connected with the affiliative field of the insula includes the frontal opercular areas GrFO, PrCO, and DO. Among them, area DO (Belmalih et al., 2009) corresponds to an architectonic sector that includes a cortical region involved in the control of voluntary facial movements (Jürgens, 2009; Coudé et al., 2011), while areas GrFO and PrCO are considered to be part of the limbic system and may act as a gateway for limbic information to enter the premotor cortex (Gerbella et al., 2014). The dorsal part of the frontal opercular region, together with the adjacent ventral premotor cortex, is known to be involved both in the production of goal-related mouth actions, including lip-smacking, and the observation of the same actions performed by others (Ferrari et al., 2003; Coudé et al., 2011). These cortical connections could be crucial for suppressing motor acts that possibly conflict with the involuntary emotional facial behavior automatically triggered by the context. The projections to the VS, which is a well-known part of the limbic basal ganglia circuit, could provide another alternative route through which the affiliative field can select and modulate a learned appropriate behavioral response and simultaneously suppress other competing emotionally or motivationally triggered motor acts (Mink, 1996).

Previous studies suggest that the frontal motor areas are part of a voluntary system exercising direct control on facial expression through the pyramidal tract and the ventral brainstem (Wild et al., 2003; Meyer et al., 2007). These authors also proposed that this voluntary system works simultaneously and independently from an involuntary or emotionally-driven system, involving subcortical structures such as the amygdala, thalamic, hypothalamic and subthalamic nuclei and the dorsal/tegmental brainstem. Our present data allow us to further extend this involuntary network to the cortex. More specifically, we suggest that the insular affiliative field, the anterior cingulate, and orbitofrontal cortex represent cortical counterparts of this emotionally-driven system, in line with a previous proposal by Gothard (2014).

Connections with the secondary somatosensory cortex and the VPMpc of the thalamus could provide a somatosensory feedback during lip-smacking movements. Accordingly, the VPMpc, albeit known mostly as a relay for taste information, could be engaged in both the sensory and motor aspects of mouth movements during lip-smacking (Norgren, 1970; Pritchard et al., 1989; Liu and Fontanini, 2014). The connections with the adjacent somatomotor hand-related insular field could play a role in the recruitment or inhibition of other emotional-based motor programs involving the insula. Finally, concerning the visceromotor system, the connections with the amygdala, the ventral tegmental area and lateral hypothalamus as well as with the anterior insula (Ongur et al., 1998; Stefanacci and Amaral, 2002; Jezzini et al., 2010) provide a neural substrate for the vegetative response (decrease in the heart rhythm) accompanying lip-smacking. It is worth noting that, in humans, hypothalamic hamartomas have been associated to the production of ictal laughter during gelastic seizures, that is, a positive-valence emotional facial expression, similar to lip-smacking (Berkovic et al., 1988; Cascino et al., 1993).

### Connectional Distinctiveness of the Affiliative Field

Functional studies indicate that the affiliative field lies adjacent to a rostral sector of the insula, involved in disgust and ingestive behaviors, and a dorsal hand-related sector extending caudally in a somatomotor forelimb/trunk field (Schneider et al., 1993; Nelissen and Vanduffel, 2011; Jezzini et al., 2012). In addition to identifying the neural network at the basis of the functional properties of the affiliative field, we aimed to assess whether this connectivity pattern is specific to this functional field by comparing its connections with those of the adjacent insular fields.

The insular disgust/ingestive field and affiliative field share similar connections to several brain areas. In particular, these two fields are reciprocally connected to emotion- and memory-related regions (orbitofrontal cortex, temporal pole, entorhinal cortex, amygdala, VS, lateral hypothalamus, and ventral tegmental area) and with sensory-motor regions (frontal opercular areas and motor cingulate area 24c). However, the disgust/ingestive field is not connected with some areas linked to the affiliative insular field, among which are the high order visual temporal areas (TE and IPa) and the hand representation of the dorsal part of the insula and of the second somatosensory cortex. The disgust/ingestive field also has distinguishing connections with the “medial prefrontal network” as defined by Carmichael and Price (1996), which include areas 14c and 13a of the “olfactory neocortex”, the medial prefrontal areas 32 and 14r, the rostralmost part of cingulate area 24a/b, and the medial prefrontal area 9. These results are in line with those of previous studies which describe the connectivity pattern of the anterior part of the insula that likely corresponds to our disgust/ingestive field (Carmichael et al., 1994; Carmichael and Price, 1995a,b; Ongur et al., 1998; Saleem et al., 2008, 2014).

Altogether these data suggest that the disgust/ingestive and the affiliative fields share some anatomical connections, in line with their common general function in generating emotional-based involuntary motor responses, but they are also driven by markedly different inputs. While the affiliative field receives visual information conveying social and contextual cues to generate a proper behavioral response (lip-smacking), the disgust/ingestive field processes somatosensory, gustatory, and olfactory inputs to formulate an emotional representation (disgust or pleasure) and drive the appropriate motor behavior (spitting or swallowing).

Our results show a clear connectional distinction between the dorsal and ventral parts of the middle insula. Specifically, only the mid-dorsal sector, but not the mid-ventral sector, corresponding to the affiliative field, projects to the hand-related parietal and premotor areas PFG and F5. These data complement the functional evidence identifying a hand-related field within this mid-dorsal region of the insula (Robinson and Burton, 1980; Schneider et al., 1993; Nelissen and Vanduffel, 2011; Jezzini et al., 2012). In this respect, our data largely extend the results described in previous studies focused on the connectivity of the insula. The results found by Mufson and Mesulam (1982) on insular connectivity failed to distinguish the dorso-ventral inhomogeneity evidenced by our anatomical and functional studies. Their injections were driven by architectonic, rather than functional criteria, and while their results appear fully compatible with an involvement of both hand-related and affiliative fields, they do not demarcate these two functional fields. Our previous ICMS studies point to a functional heterogeneity within the middle insula that had been missing from the narrative. With our current data, we show that there are anatomical distinctions in connectivity that underlie these functions and provide a more refined description of this brain area.

### Beyond Interoception: A Network for Experiencing and Expressing Emotions

The present data indicate that the affiliative field of the insula is part of a wide network whose activity represents, beside internal body states (Craig, 2010), other types of information. This additional information includes perceptual aspects of the visual scene (coded by inferior temporal areas and visual thalamic nuclei), their emotional meaning (through the amygdala and the orbitofrontal cortex), and memory of previous emotional experiences (coded by the anterior temporal pole, the perirhinal cortex, and the VS). Moreover, the convergence of this information would allow one to refine a behavioral response (cingulate cortex) by incorporating social context and suppressing conflicting motor acts (opercular frontal areas and VS). This integrative emotional processing and output would be important for generating a specific communicative motor act related to the individual’s emotional state.

The dominant view of the insula in affective neuroscience is that this region is involved in the perceptual aspects of emotion and emotional awareness. According to this proposal, the posterior insula is targeted by interoceptive input from the thalamus, which in turn projects to the anterior insula where emotional self-awareness emerges (Craig, 2009). In contrast to this view, which attributes to the insula the role of a renewed Cartesian “pineal gland” where interoceptive inputs are transformed into awareness, we suggest that the role of this region in emotions should be interpreted according to a more embodied and enactive framework, abandoning the classic distinction between emotional experience and emotional expression. Our proposal is strongly rooted in empirical evidence derived from behavioral studies in humans and monkeys. These studies show that the production of an emotional facial expression enhances the corresponding emotional experience and influences how incoming emotional cues are processed (Niedenthal, 2007). Furthermore, inhibiting the production of an emotional response at the periphery impairs emotion perception (Davis et al., 2010), and the inhibition of expressive facial feedback affects emotional experience in depressed patients (Finzi and Wasserman, 2006). Finally, Hennenlotter et al. (2009) showed that botulinum toxin injected in the frown muscles of human subjects decreased amygdalar response evoked by imitating angry facial expressions. In human studies, the role of the insula in emotional processing has been confined to perception and recognition (Kurth et al., 2010; Kelly et al., 2012), while its possible role in the production of emotional behavior has been largely underrated, mainly due to the technical difficulty in eliciting genuine emotional expressions in controlled fMRI settings. Classic stimulation studies in non-human primates have shown that the insula has the ability to drive vegetative and orofacial motor responses, even in anesthetized animals (Kaada et al., 1949; Hoffman and Rasmussen, 1953; Frontera, 1956; Showers and Lauer, 1961). Our previous ICMS studies, performed on awake monkeys in a social context, aimed at bridging the gap between these two notions of the insula (Caruana et al., 2011; Jezzini et al., 2012). Stimulating a specific sector of the ventral insula produces both an orofacial motor response (the affiliative gesture) and a congruent vegetative response (decrease in the heart rhythm), which are dependent on the establishment of mutual gaze between the monkey and the experimenter. This latter evidence indicates that the evoked behavior necessitates the integration of high-order visual and memory-related information about the identity of biological cues, the emotional aspects of the environment, and the social context of the situation. The anatomical connections between visual, sensory-motor, and emotional centers described in this paper strongly support this interpretation through identifying the specific cortical and subcortical nodes dynamically interacting during emotional expression in social contexts.

Our results challenge the classic view of the insula as a multisensory area merely reflecting bodily and internal visceral states. Together with our previous ICMS studies, we provide evidence for an alternative perspective in which the insula both processes incoming emotional cues and plays an active role in producing an appropriate emotional motor output. Our work offers data to substantiate a traditional account of emotion (Dewey, 1894, 1895) while incorporating hypotheses put forward by recent theoretical studies proposing an embodied and enactive account of emotions (Caruana and Gallese, 2012; Krueger, 2014).

## Conflict of Interest Statement

The authors declare that the research was conducted in the absence of any commercial or financial relationships that could be construed as a potential conflict of interest.
